# Mechanical Restraint in Our State Hospitals for the Insane1Read before the Montour County (Pa.) Medical Society.

**Published:** 1891-03

**Authors:** Gros. R. Trowbridge

**Affiliations:** Assistant Physician to the State Hospital for the Insane, Danville, Pa.


Buffalo Medical and Surgical Journal

VOL. XXX. MARCH, 1891. NO. 8.

### Original Communications.

### MECHANICAL RESTRAINT IN OUR STATE HOSPITALS FOR THE INSANE.[1]

BY GROS. R. TROWBRIDGE, A. M., M. D.

Assistant Physician to the State Hospital for the Insane, Danville, Pa.

SO MUCH has been said on this subject, and during the past two or three years it has been brought so forcibly to the attention of the medical staffs of our insane hospitals, that I feel almost like making an apology for saying anything in regard to it; still, I think, in the face of the present preponderance of feeling against mechanical restraint, that a few words spoken in its favor will not come amiss.

If the American public only could or would understand what is meant by mechanical restraint, as employed in our state hospitals for the insane, and the reasons for which it is used, and take a sensible and unbiased view of the matter, I am positive "exhortations and expostulations" against this "barbarous custom" would, in a great measure, cease; and could all be brought to look at the subject from a humanitarian standpoint, there would much less be said against it.

True, it is a question with which an enterprising (?) reporter of some sensational newspaper can fill a column or two, and so bring to the notice of an easily deceived public the soul-harrowing tales of the indignities and cruelties inflicted by the use of mechanical restraint in our insane hospitals.

By the word "restraint," as it is used by the alienist of today, I understand *the depriving of voluntary action of an individual either for his own or the safety of others, or for his mental, moral and physical good; the individual being irresponsible for his conduct.*

Before entering upon any discussion on this subject, let us, for the

1. Read before the Montour County (Pa.) Medical Society.

sake of a few comparisons, take a brief retrospect of the methods of restraint in vogue fifty or sixty years ago, and to accomplish this I have taken the liberty of making a few extracts from the works of the Hon. Wm. P. Letchworth, of Buffalo, N. Y., entitled "The Insane in Foreign Countries." The author, in his work, gives a clear and concise account of the methods of "restraint" used in foreign asylums half a century ago.

He says:

The methods of restraint in common use included iron hand-cuffs connected with chains to a leather waist-belt; leathern hobbles locked around the ankles, which permitted the patient to shuffle his feet, but impeded his movements so that he could not walk; iron hand-cuffs with chains, which passed through fastening locks at the sides of heavy "tub bedsteads," filled with straw, boots made of ticking, with rings and chains which passed through fastening locks at the bottom of the bedsteads.

These were methods of restraint which were at the same time unnecessary and barbarous, and are looked upon today with abhorrence and disgust. In those days the idea seemed instilled into the minds of those having the insane in charge, that these unfortunates were perfectly responsible for their acts, and the slightest outburst of maniacal excitement was treated not in a rational, careful manner, but by these relics of barbarism which fortunately for the good and welfare of the insane have gone entirely into disuse.

I may be criticised for advocating mechanical restraint and yet not upholding these methods. Still, no one could for a moment liken the restraint of today to that I have just cited. The restraint in vogue in our state hospitals is used for medical and humane reasons, while that of fifty years ago was a mere "convenience" and an easy and safe way of disposing of and caring for lunatics.

In the majority of hospitals in England, which Mr. Letchworth visited, very little restraint was in use, though all were prepared to employ it if it was thought necessary.

Of St. Anne's Hospital, in France, he says: "Restraint is used in the form of chains and bedsteads fastened to the floor," to which I suppose the patient is secured by means of straps, locks, etc.

At Gheel, Dr. Peteers has the following methods: (1.) A broad padded belt or band, five inches wide, for locking around the upper part of the arms. In this belt is an iron loop, through which passes a strap for securing the patient to the side of the bed. (2.) Two padded leather bands, three inches broad, connected in the center by a link two inches long. These are designed for the ankles of the patient. To each is attached a loop of iron, through which a strap is passed and fastened to either side of the bedstead. (3.) Two padded soft leather wristbands, three inches wide, separating the wrists about eight inches. Dr. Peteers said these were scarcely ever used. (4.) A broad leather belt encircling the body with two leather pockets in front for the hands, which are secured in their position by wristlet straps. Extending from this belt across the shoulders, suspender fashion, are straps which retain in position and relieve the weight of the belt, which locks at the back.

At none of these places was restraint used unless absolutely necessary. I have given a brief outline of the methods of restraint in some of the foreign hospitals. In this country I do not think such elaborate methods are employed, but on the contrary the appliances are much more simple and just as effective.

Of course, strong arguments can be brought to bear against mechanical restraint, but as I have said, I think these opinions are, for the most part, advanced by persons not understanding the subject. My own observations and conclusions, since my connection with a hospital for the insane, are that in the treatment of certain cases of insanity, in the control of certain individuals, it is for the good of the patient mentally, morally and physically that restraint be applied. Let me impress the fact that I do not refer to the restraint of years ago, or the reasons for employing it, but a restraint judiciously and conscientiously used under the direction of a *medical man*, who is fully competent to judge, and is perfectly familiar with the case. Some of our hospitals, in their reports, boast with evident pride that "no mechanical restraint has been used during the past year." To my mind this is no reason for a burst of enthusiasm from "non-restraint" supporters, as it only shows that, if proper care and attention was given to the inmates, either the institution contains a class of patients which does not require restraint, or else it means that in cases where mechanical restraint should have been employed, either chemical or manual restraint have been used, *i. e.*, either the patient has been overpowered by the use of strong medicines, or else has been placed in the care of several attendants, whose duty it is to see that the individual does no harm either to himself or others.

I consider manual restraint, as a rule, far more irritating and annoying to the violent and excited patient than our present mechanical methods. Is it not more aggravating to anyone, sane or insane, to be overpowered by one or more of their fellow-beings than by mechanical means? In the first case, it is a constant strain and struggle for the mastery and usually the result is the injury of either attendant or patient, or both; while in the second case a few futile attempts, and the individual perceiving that struggles are useless, will, in nine cases out of ten, cease his violence. Then, again, it is a well-known fact, that despite the most careful means of selection, not all attendants are to be trusted, and it may happen that blows are given and violence committed against the excited patient, which by mechanical restraint could easily have been avoided; besides it is a most trying task for attendants to sit hour after hour endeavoring to control an insane person who is bent on his own destruction or that of some one else, and it is not surprising that they lose their tempers and become irritable in the discharge of such a task.

Manual restraint will, without fail, cause resentment on the part of the patient, and he or she will be brought to look on the attendant as a tormentor, and this will of necessity be a detriment to a possible restoration, as it is in the power of attendants to be either a benefit or a hindrance in the care of the insane. To my mind there is no chance for a comparison between the two methods, manual and mechanical, where a safe, continued and absolute restraint is required.

I do not wish to be understood as advocating an indiscriminate use of mechanical restraint. It should be applied only by the physicians understanding the nature and necessities of the case and not by every Tom, Dick and Harry connected with the institution.

Dr. Nicolson,[1] of the Broadmoor Criminal Asylum, England, says:

The question as to the employment in lunatic asylums of what is known as "mechanical restraint," occupied a considerable amount of public attention last year. The legitimate or justifiable use of mechanical restraint is one thing, the authorized abuse of it another. The sanction of modern times in this country is clearly against the use of mechanical restraint; and very properly so in recollection of the extent to which it was carried in days gone by. The existing practice is, as a matter of fact, in accordance with this sanction; and the exceptions when this form of restraint is used for other than surgical reasons, are sufficiently few to afford the best proof of the rule. Besides sanction and practice, an element of sentiment has crept into the matter which would fain make it of the nature of a crime to use mechanical restraint at all, unless, perhaps, in surgical cases.

Mechanical restraint is practically unknown at Broadmoor. It was,

1. *Journal of Mental Science*, July, 1890.

I believe, used on a few occasions in the early days, but for over twenty years it has been found possible to do without it. The comparatively numerous staff of attendants at this asylum enables exceptional strength to be brought to bear when occasion requires it; but my own feeling in the matter is that in a case of continuous and long-sustained maniacal violence, with an activity avowedly and determinedly homicidal, it is possible to carry the non-restraint principle (so-called) too far and at too great a cost. Few people who are not engaged in it are able to estimate the wear and tear of nerve and heart, which a tussle with a desperate lunatic of this sort means to the attendants, apart from the risk of positive injury either to the patients or warders. And when this struggle has not only to be anticipated, but to be engaged in several times a day for a lengthened period, the justifiability and humanity of simple, effective and safe mechanical restraint become apparent.

If ever it should be my lot to be a lunatic, such as the one whose treatment is here in question, I hope my good friend, the superintendent, who has me in charge, will not be too squeamish about recent traditional usage, but will see that I am checked by the minimum but necessary amount of restraint to insure my own safety and that of others. On the one hand, I do not wish to think that under any circumstances I should be the means of inflicting injury upon my attendants; on the other hand, I would infinitely prefer mechanical restraint to the long continued manipulations, however skilful and friendly, of four or more attendants heaped upon my prostrate form. . . . . The evil to be feared with regard to mechanical restraint is lest its legitimate use should lead to its authorized abuse. This could never happen if, in every case, the physicians themselves were to order it to be used, and that only after a careful and complete personal investigation. When the restraint is applied by underlings and its use afterward sanctioned by the responsible officers, we have the authorized abuse which is altogether wrong and reprehensible.

The Commissioners of the Derby County Asylum, England, make the following statement:

Two females and one male have been restrained each once; the male from January 19, 1889, to February 16, 1890, to prevent self-mutilation. One woman for two nights and a day, to prevent her pulling out her hair and injuring herself, the other woman for thirty-nine hours, because she was dangerously violent to herself and others. They were all restrained by this jacket. The woman last mentioned is convalescent, and when speaking to us, remembered her restraint, and spoke gratefully of it, which appears to have been quite necessary, and to have, in fact, saved her life.

Dr. Manry Deas, of Exeter Asylum, says:

In two cases, especially the morbid impulse to self-injury was so strong, that I did not hesitate to resort, for a considerable length of time, to the employment of what is termed mechanical restraint in the form of a dress, so as to restrict the free use of the arms and hands. This I did both for the sake of the additional protection thus afforded to the patients against themselves, and also, I feel bound to say, to give some relief to the great, almost intolerable strain and responsibility devolving on those having the care of such cases. One of the patients is now convalescent and the other much improved. Restraint of some kind is the basis on which all treatment of the insane, whether legal or medical, is based ; and the enlightened modern treatment of the insane does not lie so much in the abolition of restraint as in the carrying out of various kinds of restraint, under constant and humane supervision.

. . . . . . . . . . . .

A physician having the responsible care of the insane, ought to be as free to employ mechanical restraint, when he deems it the best treatment in a particular case, as he is to use powerful drugs or any other recognized mode of treatment.

The special advantages of confining the hands by mechanical means are that the restraint is continuous while in use, it is always vigilant, it does not lose its temper, and it avoids the many risks attendant on manual restraint.

For myself, I do not hesitate to say that there are cases in which "mechanical" restraint is not only the best and most humane treatment, but in which there is a grave responsibility attaching to the man who refrains from using it. I have never regretted the use of mechanical restraint when, after full consideration, it has been resorted to ; but there have been, in the course of my experience, cases in which afterwards I regretted that I had not used it.

Such is the testimony of a few English superintendents.

The great weight of evidence brought to bear against mechanical restraint is advanced by medical men who have no idea what is meant by the term, and a large number of them who have never set foot inside a hospital for the insane, and yet they hold up their hands in holy horror at the mention of the term "mechanical restraint."

I venture to say that any of these men who are visiting physicians to our General Hospitals, never hesitate to use restraint in the "drunk" cases which are so frequently admitted to those institutions, and from my own experience as Resident Physician in a large general hospital I can testify that this restraint savors very strongly of hand-cuffs and ropes, and yet these same men consider restraint among the insane a barbarity. Perhaps, under the circumstances, they would show mercy to "crazy doctors," and at least sanction restraint when applied to alcoholic cases.

One often hears the expression, "Treat an insane man as you would a sane one."

This advice is all very well as far as it goes, but, unfortunately, it does not go very far. It is much more difficult to deal with a diseased mind than a sound one, and every physician knows that on many occasions even sane people decline to follow their directions.

It must be taken into consideration in dealing with the insane that the mind is the part disordered and makes the individual an irresponsible being, and consequently in many cases the appreciation of right and wrong, the power of reason and understanding, are destroyed, and we have the base animal nature to deal with. Let me instance two or three cases which have come under my observation. The first is that of a man who was admitted to this hospital in a most deplorable condition, both mentally and physically. Reverses in business had been the cause of an attack of acute melancholia, with extreme suicidal tendencies so strong, that he had made one attempt on his life before admission. This individual was carefully but securely restrained to a bed by means of sheets. His recovery was rapid, and four months later he was discharged, restored, and is now actively engaged in business.

I do not assert, by any means, that being tied to a bed was the source of this restoration, but I say that it was an extremely important factor in the case. The second was a case of melancholia, who, if not restrained, would disfigure himself by picking holes in his arms, and on one occasion nearly succeeded in reaching the brachial artery. Besides, his intentions were suicidal, and for these reasons restraint was applied.

The third is a case of an epileptic, imbecile boy, who, in outbursts of excitement, pounds his head on the floor with terrific force. Any manual restraint only increases his excitement, while a roller bandage, loosely applied, confining his hands behind him, has the effect of controling his violence.

Here are three cases in which restraint was justifiable, and I maintain if, as in the first of these three cases, it has been of assistance in restoring *one* individual to reason, it is a valuable aid in the treatment of the insane.

Let us consider it from another standpoint. Imagine two identical cases of maniacal excitement with violence, which makes them destructive to themselves and others. In the first case mechanical restraint has not been applied; in the second it has. Suppose both cases are restored. The first leaves the institution with scars and marks, due to his violence. The second leaves the hospital physically sound, with no marks or scars, and simply because he has worn a leather muff for a few days, or, perhaps, weeks. The first has marks of his own violence to remind him that he was at one time "crazy," while number two has not this distinction.

Again, it is not pleasant for friends to visit their unfortunate relatives and find them covered with bruises or cuts, inflicted either by their own hands or some violent patient, all of which might have been prevented by the use of mechanical restraint. Moreover, besides being an unpleasant thing for the friends of patients to see them injured in this way, it is a discredit to the hospital, though how frequently do such cases occur in all institutions for the insane!

Why is mechanical restraint a more barbarous custom than forcible feeding, and yet it is just as serious a matter to allow an individual to commit suicide by self-injury as by starvation, but one rarely hears any objection to the nasal feeding tube.

In the report of the Committee on Lunacy in this State (Pa.) for 1889, the secretary mentions the fact that there had been "no mechanical restraint" used in the female department of the Norristown State Hospital for the Insane for "the past year," commends the fact and asks "Why not the same in other hospitals?"

This inquiry is certainly a very strange one. Lunatics differ as well as sane people, and have to be treated accordingly. The female department of Norristown is to be congratulated on having such an easy class of the insane to deal with.

This matter of "mechanical restraint" should be carefully considered before it is voted a relic of barbarism.

---

Dr. Beverly Robinson stated, at a recent meeting of the Practitioners' Society of New York, that the fact had come to be pretty generally recognized that a cardiac murmur of itself meant very little. It came down to the question whether the heart was doing its work well. There were certain ways of determining that point, aside from physical signs. One evidence of lack of proper compensatory power was difficult breathing; a second was palpitation; a third was pain in and around the heart. If no renal nor other physical evidence that the heart was suffering, the mere presence of a cardiac murmur should not be considered as of great importance. — *Practice.* — *Lancet-Clinic.*

